# Effect of human pegivirus route of transmission on the genetic distribution of the virus: an institution based cross-sectional study

**DOI:** 10.1186/s12985-019-1161-5

**Published:** 2019-04-25

**Authors:** Wubet Taklual, Shixing Tang, Wu Yue

**Affiliations:** 1 Department of Population Health, College of Health Sciences, Debre Tabor University, Post office box: 272, Debre Tabor, Ethiopia; 2 Department of Epidemiology, School of Public Health, Southern Medical University, Guangzhou, China; 3 Chinese center for disease control and prevention, Beijing, China

**Keywords:** HPgV, Intravenous drug users, Men who have sex with men, Genotype, Route of transmission, China

## Abstract

**Introduction:**

Human pegivirus (HPgV), formally called GB virus C (GBV-C), is a member of the pegivirus genus in Flaviviridae family. High prevalence of HPgV infection is seen among sex workers, blood transfusion recipients and intravenous drug users (IDUs). So far, there are seven genotypes and many subtypes identified in different countries. The predominant genotype in Asia including China is genotype 3, although genotype 7 has been reported recently in China. The aim of this study was to evaluate the effect of the transmission routes of HPgV infection on the genotype distribution of the virus, to determine the prevalence rate, and identify the dominant genotype among men who have sex with men (MSM) and IDUs co-infected with human immunodeficiency virus type one (HIV-1) in Guangzhou, China.

**Methods:**

A total of 131 MSM and 70 IDUs co-infected with HIV-1 were randomly selected in Guangdong Dermatology Hospital. HPgV RNA was detected by nested reverse transcriptase polymerase chain reaction (RT-PCR) using primers. The PCR products were sequenced and phylogenetically analyzed by using MEGA6.06 version software to determine the genotypes. Chi-square and Fisher exact test were implemented for comparing the proportion between different variables.

**Results:**

The prevalence of HPgV infection was 32.9% among IDUs and 18.3% in MSM with a statistically significant difference between the two groups (*p* = 0.02). In IDU group, 82.6% infected with genotype 3 and the rest (17.4%) were categorized to genotype 7. Similarly, in MSM group, 83.3% belonged to genotype 3, and the remaining 16.7% were classified as sub-genotype 2a and 2b.

**Conclusion:**

In Guangzhou, China, the prevalence rate of HPgV infection in IDUs was higher than MSM. The dominant genotype in the two groups was genotype 3. Our results indicated that routes of transmission did not affect the genotype distribution but did affect the prevalence rate of HPgV infection.

## Background

Human PegiVirus (HPgV), formally called GB Virus C (GBV-C), is a member of pegivirus genus with in the family of Flaviviridae. It is a single stranded RNA virus and contains a single open reading frame (ORF). HPgV and hepatitis C virus (HCV) share some similar features although they belong to different genera [1–3]. So far, HPgV has been classified into seven genotypes and many subtypes. The distribution of HPgV genotypes varies across different countries of the world: genotype 1 mainly in West Africa, genotype 2 in America and Europe, genotype 3 in Asia, genotype 4 in South East Asia in particular in Vietnam, genotype 5 only in South Africa, and genotype 6 predominantly in Indonesia. Genotype 7 has recently been described in Yunnan Province, China [4–10].

Sexual contacts, injecting drugs, blood transfusion, parenteral exposure, and medical procedures have been documented as the main transmission routes of HPgV [11–15]. High prevalence rate was seen in MSM and IDUs [16–18]. Co-infection of HPgV and HIV-1 and/or HCV is common. The rate of triple infection of HPgV/HIV-1/HCV was even higher than the dual infection of HPgV/HIV-1 [4, 16].

The prevalence of HPgV varies in different countries and among the various subjects. The HPgV prevalence was reported to be as low as 0.13% among a healthy blood donors in China, whereas 88.8% of the HIV-1 positive patients were infected with HPgV in Indonesia [8, 19]. In China, high prevalence of HPgV infection was seen among IDUs and MSM [4, 9, 20].

Previous studies also indicate that HPgV prevalence is affected by route of transmission and co-infections with hepatitis B virus (HBV), HCV, and HIV-1. In addition, parenteral and sexual transmissions were the major mode of transmission of HPgV [8, 12, 17, 19, 21–26]. Although there were many studies to report HPgV infection in different population and countries, little is known whether the transmission route of HPgV affects its genetic diversity or not. Previous studies indicated that the dominant genotype of HPgV in China was genotype 3. However, in Yunnan Province, China, the majority of the IDUs infected with HPgV genotype 7 whereas, in Beijing MSM was mainly infected with HPgV genotype 3 [4, 9]. Therefore, these preliminary results may raise a question whether the routes of transmission or different risk population has an effect on the genotype distribution of HPgV. In addition, relatively higher prevalence (0.89%) of HPgV infection was reported in general population in Southern China [20]. Guangzhou is part of Southern China, but few information was available about HPgV prevalence among MSM and IDUs. Thus, the aim of this study was to evaluate the effect of route of transmission on the genotype distribution of HPgV, to determine the prevalence rate, and to identify the dominant genotypes among the two high risk populations.

## Material and methods

Institutional based cross-sectional study was conducted among MSM and IDUs infected with HIV-1, who attended in Guangdong Dermatology Hospital. A total of 131 MSM and 70 IDUs infected with HIV-1 were randomly included in the study. The participants were selected based on the screening results of HIV-1.

A structured and pretested questionnaire was completed by trained data collector in face to face interview. The questionnaires were developed and completed in Chinese language, then translated to English for analysis by another language expert personnel for its consistency.

### Ethical clearance

Ethical clearance was obtained from the Research Review Committee from Guangdong Dermatology Hospital. The participants were enrolled in the study after obtaining written consent. De-linked data and samples were used for analysis in our study.

### Blood sample collection and processing

Five microliters of blood sample was taken from each participant using vacuum tube. The plasma was separated by using centrifugation and kept at − 70 °C until analysis.

### RNA extraction

Viral RNA was extracted from 140 ul of plasma sample by using commercially available QIA amp Viral RNA mini kit according to the manufacturer instruction.

### Amplification of nested one-step RT-PCR

The Amplification of PCR was performed with primers:

- 5′-AGTGAGTTTTGGAGATGGACTGARCAG-3′ in outer forward
- 5′-GGGAAWGCYCCCCGAGCRAGCTTCCAC-3′ in outer reverse
- 5′-GTGTGAYTGCCCCAAYGGYCCCTGGGT-3′ in inner forward
- 5′- CCACARCACRAGRAACATBAGGCGYTG-3’ in inner reverse

using water as a negative control.

The first-round of PCR amplification was performed in 25ul of reaction volume. The reaction contains (PCR buffer, MgCl2, dNTP, outer forward and reverse primer, enzyme, and RNA). The reaction volume was the same as for the second-round. In the first-round 50 °C for 30 min, and 94 °C for 2 min for reverse transcription, then 35 cycles of 94 °C for 30 s,55 °C for 30 s, and 72 °C for 1 min were used for cDNA amplification. The amplification was also done for the second-round for further amplification at 35 cycles of 94 °C for 30 s, 55 °C for 30 s, and 72 °C for one min. The amplified cDNA was separated by electrophoresis with 1% agarose gel then stained with ethidium bromide substitute dye and visualized by UV illuminator. In all procedures, the standard precautions were followed to avoid PCR contamination.

All Serological markers for HIV-1, HBV, HCV, and HSV-2 were detected by using enzyme –linked immunosorbent assay (ELISA) rapid testing kit. All tests were performed according to the manufacturer instructions.

### Nucleotide sequence determination and phylogenetic analysis

The nucleotide sequences were determined by Invitrogen Company in China. Phylogenetic analysis was performed by using MEGA6.06 version software with the reference sequences, which were obtained from the GenBank of the U.S. National Center for Biotechnology Information (NCBI). Nucleotide sequence alignments were made by using CLUSTALW. The phylogenetic tree was constructed by using the maximum likelihood method under the appropriate nucleotide substitution model. Bootstrapping was performed on 1000 re-samplings of the alignments. Genotyping was implemented by using blast with at least five high homology reference sequences for each nucleotide sequence. The reference sequences used for analysis from the GenBank were; HPgV genotype 1 (AB013500, U36380, MH053117, KC618399.1, AB003291, and AB008336), genotype 2 (U63715, LT009489, D87255, AF121950, MH053116.1, MH053115.1, KP259281, AY196904.1, and U44402), genotype 3 (D90601, D87263, U75356, AF006500, D87712, AB003288, U94695, AB008335, AF017532.1, MH746815.1, and D87708), genotype 4 (AB018667, and AB021287), genotype 5 (AY949771, KP710606, KC618401.1, and KC618398.1), genotype 6 (AB003292), genotype 7 (HQ331233, HQ331234, and HQ331235), and an outlier sequence AF070476.

### Recombination analysis

Bootscanning analysis of E2 sequence was done to identify any recombination event by SimPlot version 3.5.1 using Kimura 2-parameter with a 200 base pair (bp), a 20 bp step increment, and 1000 bootstrap replicates [27]. Each E2 sequence was compared to consensus sequences generated using available GenBank references for genotypes 1 (*n* = 6), 2 (*n* = 9), 3 (*n* = 11), 4 (*n* = 2), 5 (*n* = 4), and 7(*n* = 3). Due to the limited availability of genotype 6, only reference AB003292 was used.. If > 70% of the permuted trees showed similarity to more than one genotype across the E2 region analyzed, the ‘parental’ sequences were retained within a second bootscanning analysis along with the outlier and the query sequence. The reported sequences in this study were submitted to the GenBank under the accession numbers MK686565 to MK686611.

### Statistical analysis

All statistical analyses were performed by using SPSS version 21.0 software. Frequency and percentage were calculated using descriptive statistics. Chi-square and Fisher exact test were implemented for comparing the proportion between different variables. A *p*-value < 0.05 was considered as statistically significant.

## Results

### Detection of HPgV RNA in IDUs and MSM population

To determine the prevalence of HPgV and the impact of route of transmission on the HPgV genetic diversity, a total of 201 IDUs and MSM infected with HIV-1 participants were recruited. Among them, 70 were IDUs and 131 were MSMs. HPgV RNA was identified in 47 samples. The prevalence of HPgV infection was 32.9% (23/70) and 18.3% (24/131) in IDUs and MSM, respectively. There was a statistically significant difference in the prevalence of HPgV infection between the two groups (*p* = 0.02).

### Socio-demographic characteristics and HPgV infection in the MSM

The mean age of the participant was 28.9 ± 6.1 years. More than half of them (57.3%) were between 21 and 30 years of age. Most of the participants were unmarried (87.3%). Approximately two-third of them (63.6%) had completed junior college. With regard to the occupational status of the participant, 27.8, 25.4, and 23.8% were business men, waiters, and cadres, respectively (see Table 1).

**Table 1:** Socio-demographic characteristics and HPgV infection in the MSM

Variables		No. (%)	HPgV infection	
			No. (%)	*P* value
Nationality	Han nationality	121 (92.4)	23 (19.0)	0.513
	Others	10 (7.6)	1 (10.0)	
Marital status	Unmarried	110 (87.3)	23 (20.9)	0.524
	Married	16 (12.7)	1 (6.3)	
Age	15–24	33 (25.2)	2 (6.1)	0.170
	25–34	75 (57.3)	18 (24.0)	
	35–44	20 (15.3)	3 (15.0)	
	> 45	3 (2.3)	1 (33.3)	
Educational Status	Middle school	12 (9.3)	2 (16.7)	0.669
	High school	28 (21.7)	6 (21.4)	
	Junior college	82 (63.6)	13 (15.9)	
	University	7 (5.3)	1 (14.3)	
Occupational status	Daily worker	7 (5.6)	2 (28.6)	0.714
	Farmer	2 (1.6)	–	
	Solider	7 (5.6)	1 (14.3)	
	Cadres	30 (23.8)	6 (20.0)	
	Student	13 (10.3)	–	
	Waiter	32 (25.4)	6 (18.8)	
	Business man	35 (27.8)	5 (14.3)	

In this study, the highest HPgV prevalence was recorded among participants in the age group of 45–54 years (33.3%) and those co-infected with HCV (33.3%). HPgV was often detected in Han nationality (19.0%), un-married (20.9%), completing high school (21.4%), and daily workers (28.6%). However, no statistical significant difference was obtained, probably due to the relative small numbers of participants (see Table 1).

### Phylogenic analysis and genotyping of HPgV

**Fig. 1:**
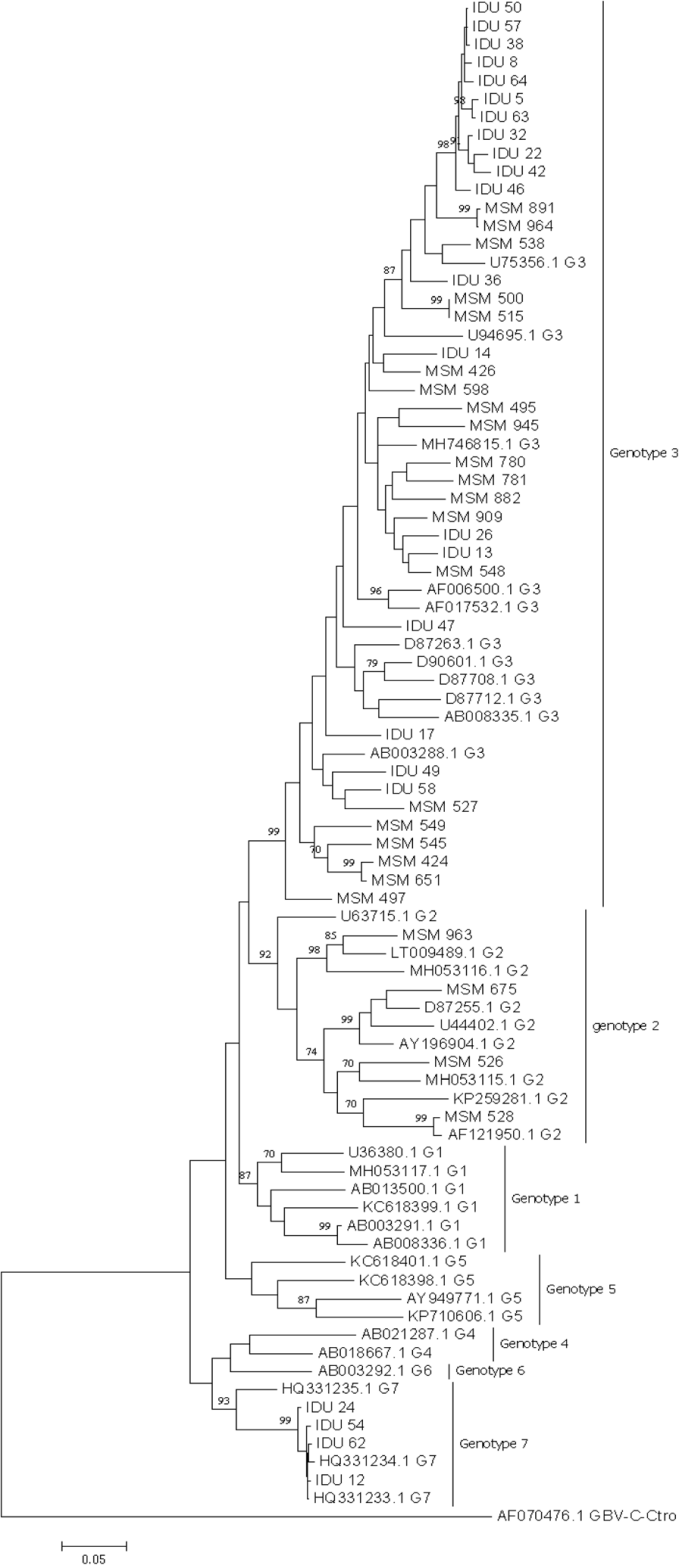
Phylogenic tree analysis was developed based on HPgV E2 fragment. Forty seven sequences identified in the current study and additional 37 reference sequences from NCBI were included. The phylogenic tree was constructed using maximum likelihood method with the GTR model. The Bootstrap analysis was performed with 1000 replications, and the bootstrap probability (more than 70%) was shown on the nodes. The scale bar represents 5% genetic distance (0.05 substitutions per site). The sequences labelled with MSM and IDU were identified in our study. The reference sequences for different HPgV genotypes were labelled with G1-G7

The Phylogenic tree was constructed using maximum likelihood with the general time reversible (GTR) model (Fig. 1). All the HPgV sequences identified in our study belong to genotype 2a, 2b, 3, and 7 (Table 2). Genotype 3 was the predominant strain. There was no statistical significance difference on the genotype distribution in both MSM (83.3%) and IDUs (82.6%) (*p* = 0.94). Furthermore, 17.4% of IDUs infected with genotype 7 whereas, 16.7% of the MSM infected with genotype 2. In this study no evidence of recombination was observed.

**Table 2:** HPgV genotype distribution among MSM and IDUs

HPgV infected-participants	HPgV Genotype (n, %)				*P*-value
	3	7	2a	2b	
MSM (*n* = 24)	20 (83.3)	–	3 (12.5)	1 (4.2)	0.947
IDUs (*n* = 23)	19 (82.6)	4 (17.4)	–	–	
Total (*n* = 47)	39 (83.0)	4 (8.5)	3 (6.4)	1 (2.1)	

## Discussion

A total of 201 IDUs and MSM infected with HIV-1 were recruited to determine the prevalence, genetic diversity, and impact of route of transmission on the genotype distribution of HPgV. In this study, the prevalence of HPgV infection in MSM and IDUs co- infected with HIV were 18.3% (24/131) and 32.9% (23/70), respectively. In our study the prevalence of HPgV among MSM is lower than that reported in previous studies [9, 17]. The difference could be explained by the sample size difference, study area, and study period. The prevalence of HPgV among IDU co-infected with HIV is consistent with other similar studies [17, 21, 22, 28].

Different studies on the prevalence of HPgV infection in different groups of population in Japan, Australia, Eastern Europe, Denmark, Italy, and Argentina showed that the prevalence of HPgV was high in MSM and IDUs than other populations [12, 13, 17, 21, 22, 24, 28–30]. These results are in agreement with the present finding. This implies that the virus could be transmitted through both parenteral route and sex contacts.

In this study, the prevalence of HPgV infection shows statistically significant difference between the two study groups (*p* = 0.02), suggesting that parenteral route may be more efficient to transmit HPgV than sex contacts. However, the difference on socio-demographic variables, the prevalence of risky behaviors, such as unprotected anal intercourse with non-regular male partners and needle sharing behavior, and the presence of other co-infections may result in higher prevalence of HPgV in IDUs than MSM.

HPgV genotyping analysis in MSM study group shows that 83.3% (20/24) of the sequences identified in our study belongs to genotype 3. The finding is consistent with other previous studies in China and Japan [29, 31–33]. The rest of the sequences coincided with genotype 2a/2b, which were commonly seen in Europe and America [22, 30, 34–36]. The distribution of HPgV genotype is in agreement with the previous study conducted in Beijing, China and Japan, in the same group of population [9, 29]. In contrast, HPgV genotype 2(2a, 2b) was rarely reported in China.

Previously in Yunnan province of China, the prevalence of HPgV infection in IDUs was 35.83% (43/120). Of which 93% were described to be infected with genotype 7 and the rest 7% were infected with genotype 3 and 4 [4] .In contrast, our finding indicated that in IDUs co-infected with HIV-1, 82.6% (19/23) were classified to HPgV genotype 3 and the rest 17.4% were categorized in genotype 7. The difference might suggest the distribution difference of HPgV genotypes in China. The finding of this study is supported by a previous studies conducted in other province of China and Japan in different group of population [9, 29, 31–33]. In this study, none of the sequences were classified as genotype 1, 4, and 6, which were more frequently found in South Africa, Vietnam and Indonesia, respectively [5, 10, 37].

This study have some limitations. First, the study was conducted in small sample size, which may affect generalizability and representativeness of the findings. Second, all the study participants were HIV-1 infected participants, and lacked some clinical information (CD4-cell counts, HIV-1 viral load, etc.) and duration of infection.

## Conclusion

The prevalence of HPgV infection was high among IDU than MSM in this study. The dominant genotype in the two groups was genotype 3, which is also predominant in Eastern Asian countries. In this study, we found that the route of transmission does not affect the genotype distribution of the virus but did affect the prevalence rate.

## Abbreviations

ELISA: Enzyme-Linked Immunosorbent Assay
GTR: General TimeReversible
HBV: Hepatitis B Virus
HCV: Hepatitis C Virus
HIV: Human Immunodeficiency Virus
HPgV: Human pegivirus
HSV: Herpes Simplex Virus
IDU: Intra-venous Drug Users
MSM: Men who have Sex with Men
NCBI: National Center for Biotechnology Information
RT-PCR: Reverse-Transcription-Polymerase Chain Reaction
SPSS: Statistical Package for the Social Sciences
STD: Sexually Transmitted Disease
